# Metabolic and behavioural effects of hermit crab shell removal techniques: Is heating less invasive than cracking?

**DOI:** 10.1017/awf.2023.17

**Published:** 2023-02-28

**Authors:** Luis M Burciaga, Guillermina Alcaraz

**Affiliations:** Departamento de Ecología y Recursos Naturales, Facultad de Ciencias, Universidad Nacional Autónoma de México, Ciudad de México 04510, México

**Keywords:** avoidance learning, crustacean, metabolism, pain, suffering, temperature

## Abstract

Hermit crabs (Paguroidea; Latreille 1802) offer great opportunities to study animal behaviour and physiology. However, the animals’ size and sex cannot be determined when they are inside their shell; information crucial to many experimental designs. Here, we tested the effects of the two most common procedures used to make crabs leave their shells: heating the shell apex and cracking the shell with a bench press. We compared the effects of each of the two procedures on the metabolic rate, hiding time, and duration of the recovery time relative to unmanipulated hermit crabs. The hermit crabs forced to abandon their shell through heating increased their respiratory rate shortly after the manipulation (1 h) and recovered their metabolic rate in less than 24 h, as occurs in individuals suddenly exposed to high temperatures in the upper-intertidal zone. Hermit crabs removed from their shells via cracking spent more time hiding in their new shells; this effect was evident immediately after the manipulation and lasted more than 24 h, similar to responses exhibited after a life-threatening predator attack. Both methods are expected to be stressful, harmful, or fear-inducing; however, the temperature required to force the crabs to abandon the shell is below the critical thermal maxima of most inhabitants of tropical tide pools. The wide thermal windows of intertidal crustaceans and the shorter duration of consequences of shell heating compared to cracking suggest heating to be a less harmful procedure for removing tropical hermit crabs from their shells.

## Introduction

Hermit crabs (Paguroidea; Latreille 1802) offer many opportunities to test biological hypotheses that are much more difficult or impossible to test in other groups of animals. Although many animals are used as models to study agonistic interactions, hermit crabs are unique. They are easy to maintain in laboratory conditions and manipulate for experimentation, they are abundant in nature, have well-defined weapons, are combative, and readily fight under laboratory conditions (Gherardi 2006). Furthermore, the primary resource that they fight over is access to gastropod shells; these shells are a discrete unit whose intrinsic and relative value as a resource are easy to assess. This usage makes hermit crabs uniquely suited for many biological studies. The shell may be a valuable tool for experimental manipulation however it can also represent something of an obstacle given that hermit crabs’ sex and size cannot be determined when they are inside the shell; information which is often deemed necessary to know at the start and/or conclusion of experimentation.

An easy, practical, and non-invasive method of motivating hermit crabs to abandon their shell and switch to an alternative (the characteristics of which are specified according to the researchers’ aims) is to attach a hair clamp to the shell. This increases the shell’s weight and modifies its centre of mass, making walking uncomfortable for the crab and most probably costly energetically; consequently, the hermit crabs ‘voluntarily’ swap to the alternative refuge provided, even when this is not ideal (e.g. too small or large; Arce & Alcaraz 2013; Alcaraz *et al.* 2020). However, there are experimental procedures which require the hermit crabs to be ‘naked’ for specific measurements or manipulations prior to starting the trials or after experimentation. Since hermit crabs’ fitness is strongly reliant upon gastropod shells, compelling them to abandon their shell requires exposure to adverse sensations or cracking of the shell to render the crabs naked. Some of the adverse sensations deployed by researchers include induction of osmotic stress by brief immersion of individuals in freshwater (Vance 1972; Scully 1979), placing them in boiling water (~100ºC; Dominciano & Mantelatto 2004), or using electric shocks of gradually increasing intensity (Appel & Elwood 2009a,b). However, the most frequently used procedures are gradual heating of the shell apex, forcing hermit crabs to abandon their shells (Fotheringham 1976a; Alcaraz & Kruesi 2009) and cracking of the shell with a bench vice (Fotheringham 1976b; Arnott & Elwood 2007).

Noxious stimuli, such as heating and stress caused by shell breakage by mechanical pressure, can affect hermit crabs in different ways. A progressive increase in temperature can bring individuals to their maximal tolerance limit, identified as a specific thermal point: the critical thermal maximum (CTMax; Cowles & Bogert 1944). Once the CTMax is reached, the individual’s physiological integrity rapidly decreases, such that animals are only able to withstand this temperature for a short time (Lutterschmidt & Hutchison 1997; Lagerspetz & Vainio 2006). CTMax values differ between various species (Beitinger & Lutterschmidt 2011).

The standardised protocol for determining CTMax involves increasing the temperature gradually until an endpoint is reached (Cowles & Bogert 1944). For swimming animals, the CTMax can be readily identifiable as a loss of the ability to maintain an appropriate position in response to the gravitation field (Becker & Genoway 1979); for benthic crustaceans, the CTMax is recognised as the temperature at which individuals lose the ability to maintain customary contact of their appendages with the substrate or the loss of righting response after balance has been lost (Lagerspetz & Vainio 2006). When individuals are returned to acclimation temperature just as they reach CTMax, they will recover their functional integrity, evidenced by recovery of the righting response (Lagerspetz & Vainio 2006). Similarly to many mobile aquatic animals, crustaceans display an escape response prior to their physiological thermal tolerance limits being reached (Tattersall *et al.* 2012); for instance, hermit crabs were seen to abandon their shells before reaching adverse conditions (overheating) when exposed to a gradual temperature increase, as previously reported for pagurids (Taylor 1982).

Shell cracking is the most frequently used procedure for forcible eviction of hermit crabs from their shells (Table 1). Since the gastropod shell is the only defensive barrier offering protection to the hermit crab’s soft abdomen (Hazlett 1981), naked individuals are extremely vulnerable to predation and damage. Therefore, shell cracking in nature frequently indicates a predation attempt. Fear, pain perception, and stressful experiences commonly generate changes in the individual’s motivational and physiological states (Elwood *et al.* 2009; Adolphs 2013; Elwood 2022); as those noxious stimuli become more intense, their effects are accentuated and become more long-lasting (Clinchy *et al.* 2013; Brown *et al.* 2015). Future behavioural decisions in animals depend upon prior experiences and information gained through past events (Denti *et al.* 1988; Daws *et al.* 2002). For instance, injuries sustained and extreme energetic demands during a long-lasting fight led to longer-lasting loser effects compared with defeats after a short fight in which no injuries were sustained (Hsu & Wolf 1999; Vieira & Peixoto 2013).

**Table 1.** Main procedures reported in the literature to evict hermit crabs from their shells

Method	Research type	References	
Shell cracking (bench press)	Animal personality	(Bridger *et al.* 2015)	(Gorman *et al.* 2018)
		(Bridger & Briffa 2015)	(Briffa *et al.* 2008)
		(Briffa *et al.* 2013)	(Mowles *et al.* 2012)
	Predation	(Briffa & Austin 2009)	(Tidau & Briffa 2019)
	Agonistic behaviour	(Arnott & Elwood 2007)	(De la Haye *et al.* 2011)
		(Arnott & Elwood 2010)	(Elwood & Glass 1981)
		(Billock & Dunbar 2009)	(Elwood & Stewart 1985)
		(Briffa & Dallaway 2007)	(Elwood *et al.* 2006)
		(Briffa & Elwood 2001)	(Lane & Briffa 2020)
		(Briffa & Elwood 2002)	(Lane *et al.* 2022)
		(Briffa & Twyman 2011)	(Mowles *et al.* 2010)
		(Briffa & Williams 2006)	(Neil, 1985)
		(Briffa & Fortescue 2017)	(Rimmer *et al.* 2021)
			(Tricarico & Gherardi 2007)
		(Courtene-Jones & Briffa 2014)	(Tricarico & Gherardi 2006)
		(Cunningham *et al.* 2021)	(Turra & Gorman 2014)
	Shell occupation and preference	(Crump *et al.* 2020)	(Fotheringham 1976a)
		(Elwood & Appel 2009)	(Fotheringham 1976b)
		(Elwood & Kennedy 1988)	(Wilber 1990)
		(Elwood *et al.* 1979)	(Yoshino *et al.* 1999)
		(Elwood *et al.* 1995)	(Yoshino *et al.* 2002)
	Physiology	(Briffa & Elwood 2004)	(Valère-Rivet *et al.* 2017)
		(Mowles *et al.* 2009)	(Velasque & Briffa 2016)
Shell heating	Agonistic behaviour	(Alcaraz & Jofre 2017)	(Grant & Ulmer 1974)
		(Burciaga *et al.* 2021)	(Hazlett 1996)
	Shell occupation and preference	(Alcaraz & Kruesi 2009)	(Argüelles-Ticó *et al.* 2010)
		(Alcaraz & Arce 2017)	(Bertness 1980)
		(Alcaraz & Kruesi 2019)	(Bertness 1981a)
		(Alcaraz *et al.* 2015)	(García & Mantelatto 2001)
		(Arce & Alcaraz 2011)	(Suárez-Rodríguez *et al.* 2019)
		(Arce & Alcaraz 2012)	(Turra & Leite 2004)
	Physiology	(Alcaraz & Kruesi 2012)	(Alcaraz & García-Cabello 2017)
		(Alcaraz *et al.* 2020a)	
Heating (Bunsen burner)	Shell occupation and preference	(Hahn 1998)	(Liszka & Underwood 1990)
Immersion in hot water	Shell occupation and preference	(Chase *et al.* 1988)	(Scully 1979)
Extraction by pulling	Shell occupation and preference	(Bulinski 2007)	(Rotjan *et al.* 2004)
Does not specify	Shell occupation and preference	(Angel 2000)	(McClintock 1985)
		(Bertness 1981b)	(Pechenik *et al.* 2001)
	Agonistic behaviour	(Bertness 1981c)	(Hazlett & Bach 2010)
	Predation	(Rittschof & Hazlett 1997)	
	Reproductive biology	(Asakura 1995)	(Wilber 1989)

Given the critical importance of the shell for hermit crabs, they are expected to abandon their shells only when exposed to stimuli that under natural contexts could lead to physical injury or death or indicate conditions that are dangerous or strongly unfavourable. Thus, it can be inferred that both heating and cracking are experienced by hermit crabs as strongly negative stimuli. Although these techniques are frequently used, their physiological and behavioural effects have not been evaluated. This study compared the functional and behavioural responses of hermit crabs removed from their shells by heating and cracking, and the recovery time required to return to baseline behaviours following each procedure. Ethically we should use the least harmful manipulations prior to behavioural experiments, but we must always consider the effects of any such manipulations on results. Recent evidence has shown that crustacean decapods, like many animals, display a relatively complex cognitive capacity and respond to noxious stimuli in ways consistent with the experience of pain (Elwood 2019; Conte *et al.* 2021). Although we cannot be sure whether crustaceans feel pain (Junaid 2015; Diggles 2019), removing the crabs from their shells induces fear-associated behaviours, so we must respect these animals’ experiences and consider their welfare.

## Materials and methods

### Ethical approval

This study protocol was approved by the Commission of Ethics and Scientific Responsibility, Faculty of Sciences, UNAM (CEARC/Bioética/0307202).

### Hermit crab capture

We captured 80 *Calcinus californiensis* (Bouvier 1898) hermit crabs of similar size occupying shells of *Nerita scabricosta* (Lamarck 1822) with no apparent damage or epibionts in Troncones, Guerrero, Mexico. The length of the chela (4–6 mm) was used as a preliminary measure to estimate the body size of the hermit crabs (Alcaraz *et al.* 2020). We kept the hermit crabs in an individual glass flask, which was also used as respirometric chamber (0.03 L), in a circulating seawater system at 27°C and 34 practical salinity unit (PSU).

### Preliminary tests

Before starting the experiments, 20 hermit crabs were removed from their shells via two separate procedures: shell heating and cracking (see below). Once the procedures were standardised, the time required to remove hermit crabs from their shells by heating (n = 10) and by cracking the shell (n = 10) was measured. These mean times were used to establish the time for the third treatment: a sham manipulation of the individuals in their shells (n = 10). Hermit crabs’ oxygen consumption and hiding times (described below) were compared among the three groups using one-way ANOVAs. We calculated the effect size (η^2^) to estimate sample size. The respiratory rate and hiding time of the individuals of the three groups differed significantly (*F*_2,27_ = 8.87; *P* = 0.001, *η* ^2^ = 0.40; *F* = 10.18; *P* < 0.001, *η* ^2^ = 0.39, respectively). The sample size was then calculated using the wp.rmanova() function of the R package ‘WebPower’ using an alpha level of 0.05, a statistical power of 0.80, and an effect size of 0.40 (Zhang 2018).

To estimate the temperature that the hermit crabs would experience when being removed from their shells using heat, the temperature increase over time in empty shells was measured. As part of standardising the procedure to force the hermit crabs to abandon their shell by heat, we measured the temperature increase of three empty wet shells following the previously standardised process. First, we drilled a 3.2-mm hole in an empty *N. scabricosta* shell on the ventral area of the apex using a Dremel multitool (Bosch Tool Corporation, Illinois, US). The shell was rinsed with seawater to remove any dust and residual material. Then, a temperature probe (Elitech GSP-6, Elitech, London, UK) was placed into the hole, with the probe tip resting on the interior wall of the shell apex, just on the other side of the shell wall from the surface where heat was applied (dorsal surface). The shell was held by the experimenter using two fingers while the tip of a hot glue gun (15 W; Truper, Mexico City, Mexico) was placed over the dorsal area of the shell apex. Even in hermit crabs that are completely retracted into their shells, most of the abdomen still remains below the upper coil of the shell (Krans & Chapple 2005) which is where we applied heat and where the temperature probe was placed. Therefore, the temperature measured by the probe is almost certainly an overestimation of that radiated throughout the rest of the shell, including where most of the individual’s abdomen resides. Furthermore, the hermit crabs’ abdomens may not be in direct contact with the inner wall of the shell. Thus, the temperature estimates represent the maximum temperature to which the individuals could be exposed. We recorded the increase in temperature (T) over time (t) measured by the temperature probe in three different nerite shells of similar size. The shells used were dried for 24 h in an oven at 60°C. Next, we compared the linear regressions of the temperature increase of the interior of the shell as a function of time (heating rates) using an ANCOVA, with shell mass as a covariate. Since the heating rate of the three shells did not differ, the resulting equation was used to estimate the maximum temperature that each individual could be exposed to before abandoning the shell as a function of the time spent heating the shell.

### Experimental groups and treatment descriptions

**Figure 1.**
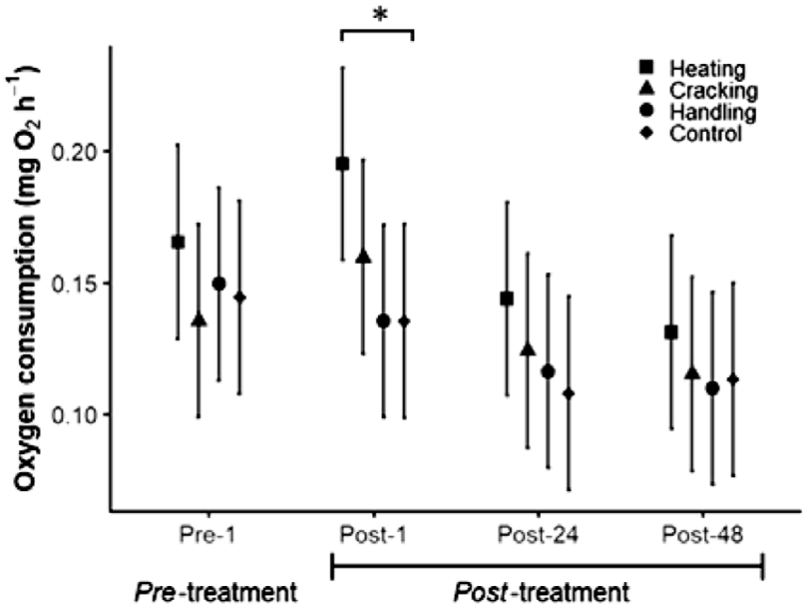
Effect of treatments on the respiratory rate of hermit crabs. Oxygen consumption before applying any treatment (1 h; Pre-treatment), and after one, 24, and 48 h of the treatment application (Post-treatment; Post-1, Post-24, and Post-48, respectively). The hermit crabs were forced to abandon their shell by heating (squares), removed from their shell by cracking it (triangles), handled by being air-exposed (circles), and unmanipulated control (diamonds). Bars represent 95% confidence intervals. Significant differences are shown with asterisks (*P* < 0.05).

One day after collection, 60 hermit crabs were randomly assigned into four experimental groups in which they were: (i) forced to abandon by shell heating (n = 15); (ii) removed by cracking their shell (n = 15); (iii) handled (n = 15); and (d) unhandled (hereafter control; n = 15; Figure 1). The crabs assigned to the shell-heating group were removed from the water with dissection forceps, then held by the shell with two fingers while the tip of a hot glue gun (15W; Truper) was placed on the dorsal area of the shell apex (see previous section). The heat was applied until the hermit crab abandoned its shell and the time taken for the crab to abandon its shell was measured (from the start of heat application until the hermit crab left the shell and all of its appendages had made contact with the experimenter’s finger). Immediately following abandonment of the shell, the naked hermit crab was placed into the water column of a container (0.04 L) that also served as a respirometer. Individuals were allowed to sink to the bottom and it was noted whether righting position was restored (appendages making contact with the substrate). Each naked crab was then immediately provided with a new, similarly sized (shell length) refuge via placement of the new shell onto the bottom of the tank with forceps.

The crabs assigned to the shell-cracking group were evicted from their shells by placing the shell in a C-clamp (4’, Truper) which was tightened gradually until a cracking sound denoted that cracking had occurred. This procedure left fragments of shell attached to the crab’s body (without apparent damage to the tissue) which the experimenter carefully removed with blunt-tip dissection forceps. The time required to crack the shell, including removing any shattered fragments (from the time the experimenter placed the shell in the clamp until the crab was fully naked and free of fragments) was recorded. As before, it was noted whether the hermit crab recovered its righting position on release into the container of water. Finally, as previously described, a new empty shell of a similar size to the original was made available.

The handling group treatment consisted of taking a hermit crab from its container and keeping it out of the water with the shell aperture (opening) facing upwards for 1 min, which was the mean time required for hermit crabs to be forced to abandon their shell via heat application. The experimenter then placed the crab in an individual respirometric chamber, where its ability to recover righting position was recorded. This experimental group allowed evaluation of the effect of manipulating individuals out of the water. Finally, the crabs assigned to the control group were not manipulated; they were kept in their respirometric chamber without being disturbed.

### Measurements before and after treatment application

The immediate and lasting metabolic and behavioural consequences of removing hermit crabs from their shells prior to manipulation and at three points following the treatment was measured. The first measurement was taken 1 h before treatment application (Pre-1) and three measurements were taken after the manipulations, 1, 24, and 48 h after carrying out the assigned treatment (Post-1, Post-24, and Post-48; shell heating, shell cracking, handling, and control; Figure 1).

#### Pre-treatment measurements (Pre-1)

The same day the hermit crabs were collected they were fed with Tetramin pellets (Spectrum Brands, Wisconsin, US) (± 10% wet weight). After 3 h of feeding any remaining food was removed with a siphon. Individuals were then placed into a clean glass container (0.03 L) immersed in a recirculated seawater system where they remained overnight. The following morning hermit crabs were carefully transferred to clean glass respirometers (0.03 L) and acclimated to the respirometer for 3 h before having their metabolic rate measured. Following this, individuals were removed from the respirometer with dissection forceps and relocated to the tank designed to measure the hiding response (see below). Then, the individuals were returned to the well-aerated recirculating seawater bath (30 L; 27°C, 34 PSU) in an individual’s glass flask, where they remained for 2 h (Figure 1).

#### Post-treatment measurements (Post-1, Post-24, and Post-48)

After 2 h, the specific treatment was applied to the hermit crab with the individual then placed in a clean respirometer with fresh aerated seawater (27°C and 35 PSU). Next, the respirometer was hermetically sealed and oxygen consumption was measured for 20 min. The hermit crab was then removed from the respirometer and placed into the experimental system designed to measure the hiding response (Post-1 measurements). Finally, the individual was placed into a clean individual container covered with a mesh immersed in the recirculating system (30 L).

Tested hermit crabs were fed for 3 h before being placed into a clean glass container in a recirculated seawater system, where they remained overnight. The following morning, the hermit crabs were carefully moved to clean glass respirometers, where they were kept for 3 h before having their metabolic rate and hiding time measured, as the previous day (Post-24; Figure 1). The next day this procedure was repeated to obtain metabolic rate and hiding time measurements 48 h post-treatment (Figure 1).

Small, magnetic stirrers were used to facilitate the water exchange with the main container during the hermit crabs’ maintenance and acclimation to the respirometer. The crabs remained in these respirometric chambers for 12–15 h prior to being measured and 1 h before taking the measurement the water was gradually changed and faeces were removed with a siphon. The procedure was repeated to measure crab responses to the different treatments at 24 and 48 h post-treatment (Post-24 and Post-48, respectively; Figure 1).

### Respiratory rate measurements

The oxygen consumption was measured using a closed respirometric system (Cech & Brauner 2011) using optical oxygen sensors connected to a Witrox 4 (Loligo Systems, Denmark). These sensors were calibrated using a solution of 1% sodium sulphite (0% saturation) and oxygen-saturated seawater (100%) at 27°C. Oxygen saturation remained over 90% during the measurements (Chabot *et al.* 2016). Dissolved oxygen concentration (mg L^−1^) underwent continuous measurement every second for 20 min and small magnetic stirrers enclosed in plastic mesh at the bottom of the respirometer were used to facilitate water movement.

The rate of oxygen consumption was estimated using the slope of the relationship of oxygen concentration as a function of time; the first 2 min of the data were discarded to avoid variation from placing the hermit crabs into the respirometer (Paschke *et al.* 2018). An empty chamber was used as a control; oxygen consumption values from the empty chamber were subtracted from the respiratory measurements of the hermit crabs (Cech & Brauner 2011). Oxygen consumption was measured during the period of lowest metabolism reported for this hermit crab species (Alcaraz & Kruesi 2012) and during the hours of low tide.

### Hiding time measurements

Hiding response times were measured immediately following the respiratory measurements. Experiments were carried out in an acrylic tank (41 × 19 × 25 cm; length × width × depth) with a mirror placed at the bottom angled at 45° relative to the horizon thereby enabling reflection of the tank floor to ascertain where the crabs placed their appendages after emerging from their shells. The hermit crabs were removed from the respirometer by hand and held for 10 s with the shell aperture facing upwards to promote complete retraction into their shell (Chávez-Solís & Alcaraz 2015). After that, the individuals were placed into the experimental tank with the aperture facing downward. Hiding time was defined as the period between placing the hermit crab on the floor and observing all its appendages making contact with the floor (Briffa *et al.* 2008). These experiments were video-recorded which helped enable quantification of hiding time via the reflection from the mirror on the tank floor.

At the end of the experiments, the crabs were extracted from their shells by heating. Cephalothorax length and weight were measured, and sexing based on the position of the gonopores took place. We weighed the shells occupied by the hermit crabs when they were collected (plate balance OHAUS [± 0.01] g; OHAUS Corporation, New Jersey, US) and we calculated the shell size fit to the individual’s body mass (Shell Adequacy Index; SAI; Asakura 1992, 1995). At the end of the experiments, the hermit crabs were provided with a new, suitable shell and returned to the collection site.

### Statistical analysis

Body masses of hermit crabs from the four experimental groups were compared using a one-way analysis of variance. It was evaluated whether the time animals took to abandon their shells when exposed to heat depended on their shell fit or sex using a linear model. Time was considered the response variable, sex as a grouping variable, and SAI as a covariate (SAI; Asakura 1992, 1995). This analysis allowed us to assess whether increased value of shells with suitable SAI increases the temperature threshold crabs are willing to endure before abandoning it.

The effect of the treatments on the respiratory rate and the hiding time were compared using linear mixed models (LMMs; lmer package in R) with different analyses performed for the metabolic and behavioural responses. For both analyses, we included the experimental group (shell heating, shell cracking, handling, and control), the time (Pre-1, Post-1, Post-24, and Post-48), and the sex (males and females) as fixed effects; we included individuals in the model as random effects (repeated measures). When significant differences were found, we proceeded to compare between groups with Dunnett’s *post hoc* test to compare the values of the groups ‘shell heating’, ‘shell cracking’, and ‘handling’ to the ‘control’ group (unmanipulated individuals); individuals were considered to have recovered from the effects of manipulation when they no longer differed significantly from the control group in Dunnett’s tests. Similar analyses were performed to test for differences in the respiratory rate and hiding time and assumptions of normality and homoscedasticity of the variances were verified using graphic exploration of the residuals. The analyses were carried out in R v.3.6.2 (R Core Team 2020).

## Results

### Preliminary tests

The heating rate of the shell was similar for the three shells tested (ANCOVA; *F*_2,24_ = 0.33; *P* = 0.72). The equation that described the heating rate was: T = 0.11 × t + 27.13 (*R^2^* = 0.97; *P* < 0.01), where T is the temperature recorded from the inner wall of the shell and t is the time spent applying heat to the shell apex. Shell mass did not influence heating rate (covariate; *F*_2,24_ = 0.62; *P* = 0.55). We used the equation to estimate the inner shell temperature when individuals abandoned it as a function of the heating time.

### Measurements pre- and post-treatment

The mean (± SEM) body size (mass) of the hermit crabs that were randomly allocated to the four treatment groups was similar (0.22 [± 0.01] g; ANOVA, *F*_3,56_ = 0.03; *P* = 0.53). The shell temperature at which the individuals abandoned their shell was not related to the Shell Adequacy Index (SAI; *R* ^2^ = 0.14; *P* = 0.75); therefore, the temperature endured by the hermit crabs was not related to the shell quality in terms of fit to body size or to their sex (*F*_1,11_ = 0.87; *P* = 0.37).

### Respiratory rate

The respiratory rate of the individuals from the four experimental groups did not differ before the treatment (Pre-treatment; Dunnett; *P* > 0.05; Table 2). In all treatments, including the unmanipulated control, mean respiratory rate decreased over time (time; LMM, *χ* ^2^_3,43_ = 91.85; *P* < 0.001; Figure 1). However, the trajectory of this change over time differed among the treatments (significant time × treatment interaction; LMM, χ^2^_9,43_ = 6.57; *P* < 0.01). The respiratory rates of the handling and the shell-cracking groups were similar to the control (unmanipulated individuals) over the course of the four records (Dunnett; *P* > 0.05); however, the respiratory rate of the shell-heating group was higher than the control group 1-h post-treatment (Post-1; Dunnett; *P* = 0.01; Figure 1). The four treatments did not affect the respiratory rates of males and females differently (LMM, *χ* ^2^_1,43_ = 6.57; *P* = 0.09).

**Table 2.** Results of Dunnett’s tests comparing the respiratory rate and the hiding time of unmanipulated (control; n = 15) hermit crabs to those exposed to three manipulation treatments: (i) forced to abandon their shell by heating (n = 15); (ii) removed from their shell by cracking it (n = 15); and (iii) handled by being air-exposed (n = 15)

		Pre-treated		Post-treated					
		(Pre)		(Post-1)		(Post-24)		(Post-48)	
	Experimental group	*t*	*p*	*t*	*p*	*t*	*p*	*t*	*p*
Respiratory rate (mg O_2_ h^-1^)	Heating	1.06	0.59	2.91	**0.01**	2.54	**0.04**	1.28	0.44
	Cracking	0.46	0.94	1.15	0.52	1.10	0.56	0.14	0.99
	Handled	0.24	0.99	0.09	0.99	0.71	0.82	0.09	0.99
Hiding time (s)	Heating	0.74	0.80	0.10	0.99	0.06	0.99	1.34	0.40
	Cracking	0.41	0.96	2.54	**0.04**	3.47	**0.003**	1.37	0.38
	Handled	0.03	0.99	0.19	0.99	0.50	0.92	1.56	0.28

Since it was the reference group, values for the control group are not shown. The metabolic and behavioural responses were measured in the same individuals over time: before being treated (Pre-treated) and 1, 24, and 48 h after the treatment (Pos-1, Pos-24, and Pos-48). The *t*-value for each Dunnett comparison test is shown; the significant differences are shown in **bold** (*P* < 0.05).

### Hiding time

**Figure 2.**
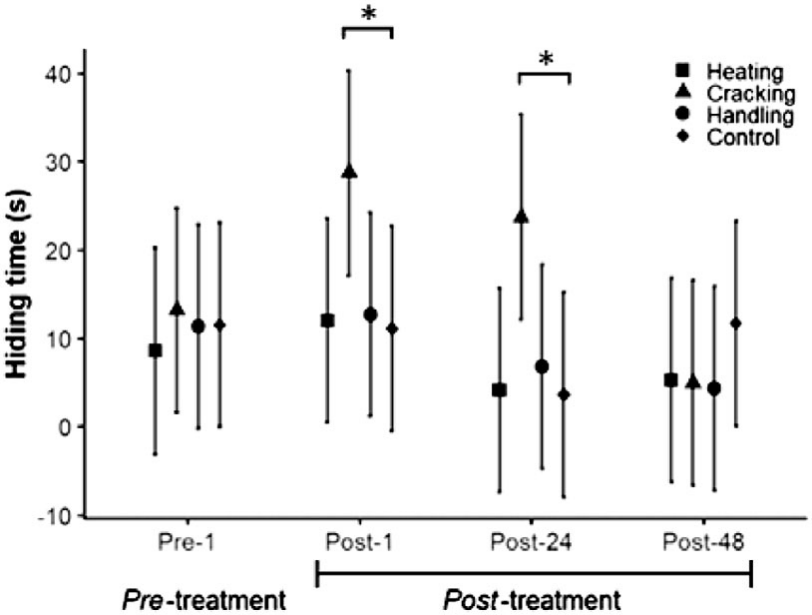
Effect of treatments on hiding time. Hiding time before applying any treatment (1 h; Pre-treatment), and after 1, 24, and 48 h of the treatment application (Post-treatment; Post-1, Post-24, and Post-48, respectively). The hermit crabs were forced to abandon their shell by heating (squares), removed from their shell by cracking it (triangles), handled by being air-exposed (circles), and control (diamonds). Bars represent 95% confidence intervals. Significant differences are shown with asterisks (*P* < 0.05).

The hiding time in response to the startle stimulus of the hermit crabs of the four experimental groups did not differ before applying the treatment (Pre-treatment; Dunnett; *P* > 0.05; Table 2). The mean time that the hermit crabs remained hidden in their shells decreased over time (time; LMM, *χ* ^2^_3,43_ = 19.54; *P* < 0.001; Figure 2) and hiding time changes differed depending on the treatment applied (interaction: time × treatment; LMM; *χ* ^2^_9,43_ = 28.22; *P* < 0.01). The hiding time of the hermit crabs in the handling group and those removed from their shell by heating were similar to the control group in all four periods (Dunnett; *P* > 0.05). In contrast, the hiding time of the hermit crabs that were removed from their shell by cracking hid for longer periods than the control 1 and 24 h after treatment (Post-1, Dunnett; *P* = 0.04; Post-24, Dunnett; *P* < 0.01). Their hiding time was similar to the control 48 h after treatment (Post-48; Dunnett; *P* = 0.49). None of the four treatments affected the hiding time response of males and females differently (LMM, *χ* ^2^_1,43_ = 6.57; *P* = 0.09).

## Discussion

Challenging short-term environmental stimuli cause changes in individuals’ physiological state that last until the functional machinery returns to the previous stable state (Ramsay & Woods 2016). Additionally, animals can respond to challenging situations by avoiding similar future events, possibly by learning, after which they attempt to avoid repeating the exposure to aversive stimuli, energetically costly activities, or dangerous situations by taking specific actions (Bateson 1991; Broom 2016). In this study, the hermit crabs responded differently to the two procedures used to remove them from their shells in ways that seem consistent with the events that each stimulus seems to resemble under natural situations. The hermit crabs’ respiratory rate increased when forced to abandon the shell by heating, but their metabolic rate returned to the previous state in a relatively short time (less than 24 h), as commonly occurs in individuals exposed to sudden peaks of temperature (*Calcinus laevimanus*; Madeira *et al.* 2018). On the other hand, the hermit crabs responded to shell cracking by increasing hiding time after a startle stimulus, and this effect lasted at least 24 h after the treatment application, similar to the expected response after a predation attempt (Brown *et al.* 2020).

The body temperature and metabolic rate of ectothermic animals are positively correlated, and both are determined primarily by the environment (Watling 2013). The oxygen capacity limitation of thermal tolerance hypothesis (OCLTT) proposes that when temperature increases rapidly, the oxygen demand exceeds the capacity of the cardiorespiratory and ventilatory systems to meet physiological demand (Pörtner 2010; Verberk *et al.* 2016). Thus, the increase in oxygen consumption by the hermit crabs forced to abandon their shell by heating can be explained as a direct consequence of the heat applied during the procedure. Particularly in hermit crabs, the exposure to a linear increase in temperature causes an increase in oxygen consumption and activity levels and induces individuals to protrude from the shell aperture as an adaptive response to adverse conditions (Taylor 1982; Wada *et al.* 2010). However, a progressive increase in temperature that exceeds the individual’s physiological limit (ie beyond the temperature at which righting response is lost) can compromise its physiological integrity, leading to irreparable functional damage unless the temperature decreases (Cowles & Bogert 1944; Lagerspetz & Vainio 2006; Beitinger & Lutterschmidt 2011). However, all of the hermit crabs forced to abandon their shell by heating righted themselves and placed their appendages on the substrate immediately after reaching the bottom of the flask. These behavioural responses are evidence that the individuals were exposed to temperatures below their critical thermal limit (Lagerspetz & Vainio 2006). Accordingly, although the oxygen consumption increased after removing the crabs from their shell by heating them (1 h), individuals recovered their metabolic rate in less than a day.

Similarly to other intertidal macroinvertebrates, hermit crabs can overcome acute exposures to high temperatures (Tomanek & Somero 1999; Lagerspetz 2003) and acclimate to them within a few hours as an adaptive response to the drastic physical changes caused by tidal rhythm (Nagabhushanam & Sarojini 1969). The thermal tolerance of intertidal hermit crabs is high, especially in species that inhabit the upper and medium intertidal zones (Taylor 1981; Turra & Denadai 2001; Kasuya *et al.* 2020). For instance, the Critical Thermal Maximum (CTM) of the tropical hermit crab (*Clibanarius albidigitus*) is 41.5°C (Vinagre *et al.* 2018). The mean estimated temperature of the interior shell at which the hermit crabs abandoned their shells was 38°C (98 s). However, hermit crabs wrap their abdomen around the columella while they are retracted into their shell (Krans & Chapple 2005); thus, the hermit crabs’ abdomen is unlikely to touch the inner wall of the last coils of the shell’s apex where we measured the temperature. This fact, and the complete recovery of the righting response and equilibrium, indicate that *C. californiensis* abandons its shell at temperatures below its thermal limit.

A potential inconvenience to removing the crabs from their shell via heating is that individuals could place different values on different shells, and therefore be willing to endure a more noxious stimulus to retain it (Appel & Elwood 2009b). For instance, the hermit crab (*Pagurus bernhardus*) shows a motivational trade-off associated with the shell quality; individuals in the preferred shell abandoned it at a higher shock intensity than those in poor quality shells (Appel & Elwood 2009b; Elwood & Appel 2009). However, in *C. californiensis* the temperature endured by the individuals was not related to the shell quality, at least in terms of shell fit.

Cracking the shell using a bench press is the other most frequently used procedure to remove hermit crabs from their shell. Fracturing the shell did not affect the crabs’ respiratory rate. Instead, the crabs removed from their shells through cracking spent more time retracted inside their new shell after the shell was cracked relative to untreated individuals; the ones manipulated out of the water, and those forced to leave the shell by heating. Long-lasting motivational changes enable the animal to avoid similar potentially dangerous or harmful situations in the future (Bateson 1991; Elwood *et al.* 2017); the greater the noxious stimulus, the more long-lasting the future impact. For instance, individuals suffering injuries during a contest show a loser effect for longer (Hsu *et al.* 2006; Briffa & Sneddon 2007; Okada *et al.* 2019). The experience of shell fracture could resemble a life-threatening predator attack, since once their shell is broken the hermit crabs have no other defence to survive a predation attempt. If so, it should not be surprising that, after their shells were cracked, the hermit crabs increased the time spent hiding in their new refuge and that this response lasted for more than 24 h. Alternatively, the hermit crabs could have remained hidden for longer as a camouflage response to reduce the risk of being exposed to another predation attempt in an area where they perceive the risk to be high.

An interesting response was the decrease in metabolic rate during the time the hermit crabs remained in the laboratory. After moving from the field to laboratory conditions, hermit crabs have shown variation in several functional and behavioural responses. For instance, *C. californiensis* pay a high metabolic cost for using heavy shells in the wild, but after several days under more amenable controlled laboratory conditions, their metabolism recovers and no longer differs from those occupying light shells (Alcaraz & Kruesi 2012). Meanwhile, the hermit crab (*Clibanarius vittatus*) hides longer in its shell after being startled under laboratory conditions compared to individuals in the wild (Gorman *et al.* 2018). Our results highlight the relevance of considering the motivational and physiological changes in captivity versus in the field, and caution is required when extrapolating the results of laboratory experiments to wild animals.

Although several animals present different behavioural or physiological responses to noxious stimuli depending on their sex (e.g. humans: Riley *et al.* 1998; rats: Cairns *et al.* 2001; Craft *et al.* 2004; and crustaceans: Appel & Elwood 2009a), we found no differences between males’ and females’ responses to any of the experimental treatments. This is similar to findings in *P. bernhardus*, in which there are no sex differences in the probability of leaving the shell due to electroshocks (Magee & Elwood 2013).

Here, we show that the metabolic and behavioural effects of evicting hermit crabs from their shells by heating and cracking dissipate in 24 and 48 h, respectively. Thus, neither shell heating nor cracking have prolonged effects. It is important to consider these times when designing experimental procedures or releasing hermit crabs after temporary captivity; before these times have elapsed, further experimental manipulations could be subject to biases, and releasing hermit crabs into the wild could increase their vulnerability to natural challenges.

Both procedures can be used to remove hermit crabs from *Nerita* or *Littorina* shells (with relatively thin walls). Forcing hermit crabs to leave their *N. scabricosta* shells by cracking is a relatively fast and straightforward procedure, in part because *Nerita* gastropods are considered to have the thinnest shell walls among those most frequently used by *C. californiensis* (Arce & Alcaraz 2011; Chávez-Solís & Alcaraz 2015). However, tropical gastropods can have thick shells due to increasing calcium carbonate saturation with increasing water temperature and as an adaptation to the higher predator pressure relative to the lower latitudes (e.g. *Littorina obtusata*: Trussell 2000; Trussell & Smith 2000; Watson *et al.* 2012). In our experience, the cracking procedure can frequently fail when applied to thick shells because some sharp portions can detach and readily damage the hermit crabs’ soft abdomen. Nevertheless, temperate regions are exposed to colder water and lower daily and seasonal temperature variations, so they could also have narrower thermal windows (Vinagre *et al.* 2018; Ángeles-González *et al.* 2020) which could lead to different effects of shell heating. A similar study comparing the shell-heating and cracking procedures could shed light on the consequence of each of these procedures as a function of the hermit crabs’ habitat. Tropical tide pools can reach extremely high temperatures during periods of low tide that prevail until the next tide. Indeed, Vinagre *et al.* (2018) found that tropical tide pools can even function as refuges for intertidal species that are able to tolerate temperatures above the upper thermal limits of their predators. Given this fact and the shorter duration of the consequences, we prefer the shell-heating method to remove hermit crabs from their shells.

### Animal welfare implications

The level of stressful experiences determines the strength and retention of their consequences (e.g. level of predatory risk; Brown *et al.* 2015). Recent investigations have shown that invertebrates, like other animals, can experience unpleasant sensations and fear-associated behaviours that can be prolonged in memory (Adolphs 2013; Appel & Elwood 2009a,b). If the more prolonged physiological and behavioural alterations after the stimulus indicate higher levels of pain, negative, or adverse sensations, heating to remove the hermit crabs from their shell should be used, at least when they are in thin-walled shells in tropical areas. The protection and welfare of the animals used for experimentation or any other purpose (e.g. aquaculture, food, industry) must be thoughtfully considered in the decision-making related to their management. Shell heating appears to be less stressful than shell cracking but still causes stress to the animals. Since hermit crabs demonstrate signs of stress in response to both methods of removing them from their shells careful consideration should be given to using these procedures. Harm-benefit studies should be carried out to consider whether the research’s benefits outweigh the harmful effects of shell removal.
